# Semantic Radical Consistency in Incidental Chinese Vocabulary Learning: A Comparison of Native Readers and Chinese as a Foreign Language (CFL) Learners

**DOI:** 10.3390/bs16081317

**Published:** 2026-08-03

**Authors:** Fei He, Shaobei Xiao, Kanghui Cheng, Yanwei Yang, Ruitai Cao, Xuejun Bai

**Affiliations:** 1 School of Psychology, Hainan Normal University, Haikou 571158, China; xiaoshb@hainnu.edu.cn (S.X.);; 2 Adolescent Psychological Development and Education Center of Hainan, Haikou 571158, China; 3 Key Research Base of Humanities and Social Sciences of the Ministry of Education, Academy of Psychology and Behavior, Tianjin Normal University, Tianjin 300387, China; baixuejun@tjnu.edu.cn; 4 Faculty of Psychology, Tianjin Normal University, Tianjin 300387, China; 5 Tianjin Social Science Laboratory of Students’ Mental Development and Learning, Tianjin 300387, China

**Keywords:** incidental vocabulary learning, semantic radical consistency, Chinese reading, eye movement, Chinese as a foreign language (CFL)

## Abstract

Semantic radicals are meaning-bearing sublexical components of Chinese characters that have been shown to facilitate incidental vocabulary learning in L1 Chinese readers. However, it remains unclear whether this sublexical mechanism extends to learners of Chinese as a foreign language (CFL), and how semantic radicals shape the online processing and learning outcomes of novel words in both populations. The present study investigated the role of semantic radical consistency in incidental vocabulary learning among L1 readers and CFL learners. Eye movements were recorded while participants read sentences containing novel two-character pseudowords whose semantic radicals were manipulated to be either consistent or inconsistent with the word’s semantic category. Both groups successfully acquired orthographic and semantic representations of the novel words, with L1 readers outperforming CFL learners. Eye-tracking data revealed a significant group-by-consistency interaction during initial exposures. L1 readers exhibited dynamic sensitivity to radical consistency, allocating more fixation time to inconsistent items at first exposure and rapidly adjusting by the second, whereas CFL learners showed no reliable online consistency effects. Moreover, reading time proved a task-specific predictor of learning outcomes. Longer initial fixation benefited orthographic learning, while extended cumulative reading time was negatively associated with semantic accuracy. Taken together, these findings suggest that sublexical semantic cue deployment during real-time reading is qualitatively distinct between L1 readers and CFL learners.

## 1. Introduction

### 1.1. Incidental Vocabulary Learning and Sublexical Morphological Cues

Vocabulary acquisition forms the bedrock of reading proficiency, in a process that extends across a lifetime (Gaskell & Ellis, 2009). Once readers accumulate enough vocabulary to navigate texts independently, incidental vocabulary learning, which refers to picking up new words as a by-product of reading for comprehension, takes over as the engine of vocabulary growth in naturalistic everyday reading (Nagy et al., 1985). Consistent with this definition, incidental vocabulary learning is characterized by the absence of an explicit learning intention rather than the absence of contextual support. As long as learners’ primary attention is directed toward comprehension rather than toward memorizing word forms, vocabulary knowledge that accrues during reading qualifies as incidental (Hulstijn, 2003; Nation, 2022). This process responds to multiple influences, including the novel words’ exposure frequency (Yanagisawa & Webb, 2021), length (Lowell & Morris, 2014), and sublexical morphological transparency (Brusnighan & Folk, 2012), alongside contextual informativeness (Lowell et al., 2020) and individual differences in spelling ability, vocabulary acquisition efficiency, and developmental stage (Eskenazi et al., 2018; Liang et al., 2021; Xiang et al., 2025). Among these factors, sublexical morphological transparency has garnered particular attention, as morphemes in alphabetic languages and semantic radicals in Chinese provide systematic cues to word meanings. Recent evidence has demonstrated that semantic radical transparency facilitates incidental vocabulary learning during Chinese reading, with this effect modulated by individual differences in processing strategies (He et al., 2026; Xiang et al., 2025). The following sections review the theoretical and empirical foundations that motivate the present study.

The focus-enrichment model (Rieder, 2002) provides a theoretical framework for understanding incidental vocabulary learning, suggesting that success depends on directing attention to novel words and enriching their semantic representations through contextual inference. This process unfolds incrementally across multiple exposures, as readers integrate contextual information with internal word cues, including sublexical components such as semantic radicals, to construct form–meaning connections (Nagy & Scott, 1990; Webb, 2019; Zhang & Li, 2016). Such incremental learning typically exhibits a characteristic trajectory; rapid initial improvement followed by a plateau, as documented in studies employing six to twelve exposures (H. S. S. L. Joseph et al., 2014; H. Joseph & Nation, 2018; Liang et al., 2021; Pellicer-Sánchez, 2016; Tamura et al., 2017). Eye-tracking, as a direct measure of attentional focus, has been instrumental in unpacking this trajectory. A key finding is the “familiarity effect”; fixation durations on novel words have been shown to decline most sharply within the first three to four exposures (Elgort et al., 2018; Godfroid et al., 2018; Pellicer-Sánchez, 2016). This effect underscores the critical importance of the initial encounters, with first-exposure processing proving highly diagnostic of ultimate learning outcomes (Borovsky et al., 2012; Share, 2004; Zhang & Li, 2016). Eye-tracking research thus provides robust empirical support for the theoretical postulate that selective attention to novel lexical forms is fundamental to incidental learning (Nation, 2022).

### 1.2. Semantic Radicals in L1 Chinese Reading

The influence of sublexical morphological information on word recognition and vocabulary learning is a cross-linguistic phenomenon. Evidence from alphabetic languages indicates that morphological transparency aids the processing of novel compound words (Brusnighan & Folk, 2012). This principle of morphological facilitation, however, operates within a different architecture in Chinese, which is specifically a morphosyllabic system where over 80% of 7000 commonly used characters are semantic–phonetic compounds. In this system, each character integrates a semantic radical (cuing meaning) with a phonetic component (suggesting pronunciation). Consequently, Chinese reading relies less on consistent form–sound mapping, which is relatively irregular (Chen & Shu, 2001), and more on direct orthographic access to semantics at the sublexical level. The semantic radical serves as the cornerstone of this direct access. Although its predictive reliability is not absolute (with overall semantic transparency estimated at around 44% (Kang, 1993), its functional utility is substantial. Empirical studies show that learners can leverage these radicals to infer word meanings with accuracy rates exceeding 60% (C. Williams & Bever, 2010). Semantic radicals thus provide a critical and comparatively efficient route to lexical meaning, making Chinese an ideal testbed for examining whether readers utilize sublexical semantic cues during incidental learning and how such engagement unfolds over repeated exposures.

A well-established body of research confirms that semantic radicals facilitate reading processes in Chinese, from character recognition (Feldman & Siok, 1999) and semantic judgment (C. Williams & Bever, 2010) to parafoveal preview (Yan et al., 2012). This reliance on semantic componential cues becomes especially critical for low-frequency characters, where readers strategically shift from holistic to component-based analysis (Taft, 2006; L. Zhou et al., 2013). This component-based strategy follows a developmental path in native Chinese readers. Young children initially show a reliance on phonetic cues when analyzing characters (M. Li et al., 2021), but as their literacy grows, this reliance undergoes a shift toward a greater weighting of semantic radical information (Tong et al., 2017), with awareness of semantic radical functions emerging by third grade and increasingly informing character learning thereafter (Ho et al., 2003; Y. Li et al., 2020a, 2020b). Since novel words do not have established lexical representations in the mental lexicon, their cognitive processing resembles that of words with extremely low frequency (Chaffin et al., 2001). Readers need to engage in analytical processing to decode form–meaning correspondences and construct semantic representations of new vocabulary. Recent studies of eye-tracking and incidental vocabulary learning have revealed how semantic radicals affect online processing. He et al. (2026) found that adult readers made fewer regressions to novel words with transparent (vs. opaque) radicals during initial learning exposures. Xiang et al. (2025) added nuance, showing that this advantage of semantic radical transparency was pronounced and stable across multiple exposures, but primarily among highly efficient readers. For native readers, automated sublexical semantic decoding appears to be both a robust strategy for vocabulary acquisition and a potential marker of reading proficiency.

Three related terms warrant clarification before proceeding. Semantic radical transparency denotes the degree of semantic correspondence between a radical and the meaning of an existing character—a continuous variable applicable to characters with established lexical representations. Semantic radical consistency is used in the broader literature as a general descriptor of a radical’s reliability in cueing meaning. In the present study, which employed novel pseudo-characters without lexical representations, conventional transparency ratings are inapplicable; the key manipulation is therefore operationalized as semantic radical–category consistency: whether the superordinate semantic category signaled by the radical (e.g., 虫 → insect) aligns with the superordinate category implied by the sentence context (Wang et al., 2026). This term is used consistently throughout this paper. This well-characterized profile of native processing frames a critical unanswered question for the field of CFL acquisition: how do these effects of semantic radical consistency operate in non-native learners?

### 1.3. Semantic Processing in CFL Learners

Whether non-native learners similarly utilize sublexical semantic information during incidental word learning remains an open question. Existing studies reveal a complex pattern. Theoretical frameworks like the non-native Chinese character processing (NCCP) framework (Tong et al., 2016; Tong & Yip, 2015) propose that CFL learners may develop a semantically driven processing pathway (C. Williams, 2013), as semantic radicals offer more reliable meaning hints than phonetic components. Offline character learning studies support this view, indicating that teaching semantic radicals helps non-native Chinese speakers infer the meanings of unfamiliar characters in sentence reading (Nguyen et al., 2017). Complementing this, Zhang and Li (2016) demonstrate that morphological awareness significantly predicts intermediate-level learners’ success in form recognition and inference of meaning during incidental character learning, whereas no such predictive relationship emerges for advanced learners, who instead rely more heavily on contextual and syntactic cues to construct meaning. This tendency to rely on sublexical meaning cues aligns with findings from alphabetic L1 learners, who depend more on semantic radicals, not due to phonological deficits, but because these components offer consistent meaning cues and are emphasized in instruction. Although phonetic radicals are designed to indicate pronunciation, the historical evolution of the Chinese writing system has rendered many such correspondences unreliable. Fewer than 40% of phonetic components provide valid phonological cues for the characters in which they appear (X. Zhou et al., 1999; Pollatsek et al., 2000), making the orthography-to-phonology route considerably less dependable than the orthography-to-semantics route (X. Zhou et al., 1999; Ke, 1998; Shen & Ke, 2007).

However, online processing studies complicate the picture. A recent study of eye-tracking and character processing revealed that intermediate CFL learners exhibited a phonetic bias, allocating significantly more attention to phonetic radicals than to semantic ones (Wood et al., 2025). This divergence between offline strategic behavior in character learning and recognition tasks and online spontaneous attentional bias in eye-movement studies suggests that the deliberate semantic processing observed in controlled tasks may not seamlessly carry over to incidental learning during reading. Crucially, existing evidence relies primarily on offline measures, which capture learning outcomes but not real-time cognitive process. A key methodological gap thus remains; we still lack a paradigm that can directly link real-time eye-movement signatures of sublexical semantic cues to immediate learning outcomes in non-native readers.

### 1.4. Eye-Movement Evidence and Proficiency Effects

This gap becomes particularly pressing given that eye-movement patterns are shaped by reading development and language proficiency. According to the linguistic proficiency hypothesis (Reichle et al., 2013), differences between eye-movement patterns stem primarily from lexical processing efficiency and post-lexical integration, not from oculomotor control per se. The evidence is straightforward; lower orthographic knowledge drives longer fixations; slower lexical processing triggers more regressions (Mancheva et al., 2015). Pellicer-Sánchez (2016) illustrated this by tracking familiarity effects across eight exposures in both L1 and L2 readers. Both groups improved at similar rates, yet L2 readers needed more encounters, typically by the third or fourth exposure, before showing the reductions in fixation durations that L1 readers achieved earlier. The critical difference, therefore, lies not in ultimate learning capacity but in processing efficiency. Recent work on Chinese word segmentation further complicates this picture: L. Li et al. (2025) found that moderate- to high-proficiency L2 readers showed less selective reanalysis than native speakers, holding onto multiple candidate interpretations longer and struggling to suppress contextually implausible alternatives, particularly in late-stage measures such as total reading time and regressions. Whether this proficiency-related constraint extends to semantic radical processing during incidental vocabulary learning remains an open question.

### 1.5. The Present Study

Guided by the above gap, the present study addresses two questions.

**RQ1:**  *Do native Chinese readers and non-native readers differ in exploiting semantic radical–context consistency during incidental vocabulary learning, and how do these differences unfold across learning exposures as indexed by fixation durations and regression probability?*

**RQ2:**  *How do group differences in online processing relate to immediate post-reading learning outcomes in orthographic and semantic knowledge?*

RQ1 refers to whether sublexical semantic processing represents a universal strategy driven by orthographic structure or a proficiency-dependent strategy that strengthens with experience; RQ2 addresses whether real-time processing signatures predict the depth of incidental form-meaning acquisition.

The following hypotheses were tested: (1) both groups would show familiarity effects, reflected in progressive reductions in fixation time across exposures, with CFL leaders overall exhibiting longer fixations and higher regression probabilities; (2) semantic radical consistency would facilitate semantic learning outcomes more strongly than orthographic outcomes, given the direct role of radical cues in meaning inference; (3) the magnitude and time course of consistency effects would differ between groups, with native readers showing larger effects during initial exposures and CFL learners showing attenuated effects given their less automatized sublexical processing; (4) fixation durations during initial exposures would positively correlate with orthographic learning outcomes, indicating the role of early visual–orthographic encoding.

## 2. Materials and Methods

### 2.1. Participants

The participants were 31 native Chinese readers (*M*_age_ = 20.45 years, *SD* = 1.34; 27 females) and 28 CFL learners (*M*_age_ = 23.29 years, *SD* = 2.80; 18 females), all university students. All CFL learners had achieved at least HSK Level 4 (a standardized six-level proficiency test administered by the Chinese Ministry of Education). Their average HSK proficiency was total score 228.58/300 (*SD* = 31.30), listening 80.68/100 (*SD* = 11.55), writing 72.32/100 (*SD* = 13.13), and reading 78.08/100 (*SD* = 16.99), indicating moderate to high Chinese proficiency. All participants were naive to the existence of the post-test phase. Participants were compensated upon completion of the experiment; compensation was performance-contingent, reflecting task engagement quality, and this arrangement was communicated to all participants in advance via the recruitment materials.

To assess the participants’ Chinese language proficiency, three measures were administered: the LEXTALE test (a lexical decision task for vocabulary knowledge), a vocabulary knowledge test (proportion correct), and a radical awareness test (assessing knowledge of semantic radicals in Chinese characters). Mann–Whitney U tests revealed significant differences between native Chinese readers and international CFL learners across all three measures (see Table 1 for descriptive statistics and test results). Given the documented facilitative role of semantic radical knowledge in learning novel Chinese words and semantic inference, radical awareness scores were included as a covariate in the linear mixed-effects models (LMMs).

### 2.2. Experimental Design

We adopted a mixed factorial design with three factors: 2 (reader group: L1 readers vs. CFL learners) × 2 (semantic radical–category consistency: consistent vs. inconsistent) × 9 (exposures: 1–9).

### 2.3. Experimental Materials and Tasks

#### 2.3.1. Materials of Learning Phase

(1) Novel words and contexts

Following He et al. (2026), sixteen novel words were constructed, each comprising two orthographically legal pseudo-characters that shared the same semantic radical and had left–right structure (e.g., 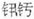). To ensure that the two experimental conditions differed only in semantic radical–category consistency, we implemented the following controls: (i) Phonological cues were minimized by selecting the right components of pseudo-characters from uncommon characters listed in The Unicode Consortium (2020). Sixteen such components were used as the right-side parts across all items. (ii) Visual complexity was equated across conditions; there were no significant differences in stroke counts for the first (8.13 ± 1.46) and second (8.50 ± 1.20) pseudo-characters of the target novel words between the two conditions (*t*(7) = −0.53, *p* = 0.61; *t*(7) = −0.18, *p* = 0.86) (see Supplementary Information and Appendix A).

Sixteen novel words were organized into eight semantic categories (mammals, vehicles, metal products, insects, female occupations, food, soil, and plants), with two words per category. Each word was embedded in nine informative sentences, yielding a total of 144 sentences. Sentence length ranged from 12 to 31 characters (*M* = 18.69, *SD* = 3.43). Depending on whether the semantic radical’s categorical meaning aligned with the category implied by the sentence context, we formed two conditions: (1) consistent, e.g., a novel word with the radical “虫” (insects, e.g., 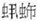) described as an insect; (2) inconsistent, e.g., a novel word with a non-matching radical “
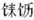
” described as an insect. To ensure accessibility for CFL learners, vocabulary and character difficulty were controlled to HSK Levels 4-5 or below, based on the *Chinese proficiency grading standards* (Center for Language Education and Cooperation, Ministry of Education of the People’s Republic of China, 2021a, 2021b, 2021c). The target novel words were embedded in the middle of sentences and were positioned to avoid being flanked by high-frequency function words (e.g., “的”) (Zang et al., 2018). All 144 experimental sentences are available as Supplementary Material (PDF file). The semantic radicals used were authentic components drawn from real Chinese characters rather than invented forms; the pseudo-characters as whole forms do not exist in any Chinese dictionary, and their meanings were constructed entirely through the experimental learning contexts rather than through explicit instruction. Participants were not informed of the novel words’ meanings prior to or during the experiment; instead, meaning was expected to be inferred incidentally from the contextualized sentences. At the end of the session, participants were debriefed and informed that the pseudo-characters had been constructed specifically for the purposes of the experiment.

To assess the fluency and complexity of the learning sentences, we conducted a pilot test with a separate group of 49 university students who did not participate in the formal experiment. Participants rated sentence fluency and difficulty on 5-point scales (higher scores indicating greater fluency or greater difficulty, respectively). To mitigate the potential effect of pseudowords on ratings of sentence complexity and coherence, we followed the approach of Liang et al. (2015) by replacing the novel word within each sentence with a semantically equivalent word from the same semantic category. For example, in the sentence “After 30 days of growth, those white 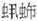 will be able to learn to fly,” the pseudoword was replaced with “silkworms” prior to rating. Results indicated that the sentences were rated as fluent and easy to understand, with difficulty scores falling in the lower range and fluency scores in the upper range of the 5-point scales (*M*_difficulty_ = 1.85, *SD* = 0.92; *M*_fluency_ = 4.17, *SD* = 0.90). Additionally, three yes/no comprehension questions were randomly distributed across the nine learning sentences for each novel word to ensure that participants attended to meaning during the learning phase. Table 2 presents representative learning sentences.

(2) Semantic activation materials

**Figure 1:**
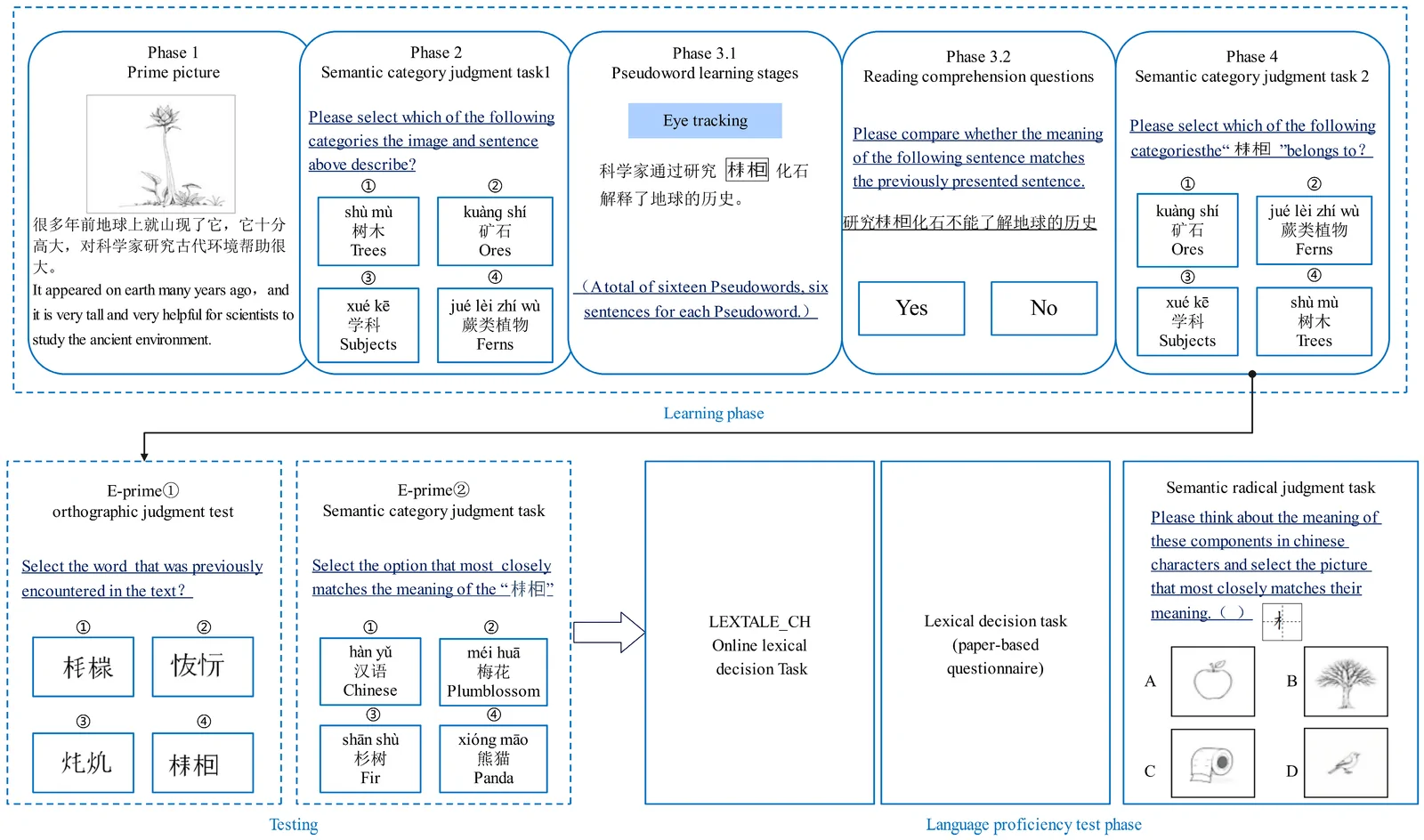
Diagram of the experimental procedure. Note: The two Chinese example sentences presented in the figure are translated as follows: Phase 3.1: “Scientists explained the history of the Earth by studying ‘
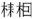
’ fossils.” Phase 3.2: “Studying ‘
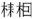
’ fossils cannot help us understand the history of the Earth.” The pseudo-words (e.g., “
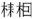
” and other novel words) shown in the figure are artificial experimental stimuli with no semantic content in any language. They are constructed for the purpose of the learning task and do not correspond to any real Chinese characters or words. Therefore, they are not translatable.

Semantic activation materials were presented prior to the learning sentences for each novel word to support the construction of semantic representations and to approximate CFL learners’ common learning modes. Each set comprised an AI-generated image and a background sentence that described the concept without using the novel word (see Figure 1, top-left panel). The images were generated using Stable Diffusion AI and were designed to depict a specific imaginary individual entity, analogous in concept to the Chinese dragon, which amalgamates features from multiple real animals into a single fantastical creature, rather than a generic categorical illustration. This design ensured conceptual correspondence between the image and the novel pseudo-character, both of which referred to an entirely novel, non-existing referent. Critically, although each image depicted a specific imaginary individual, it was designed such that its superordinate categorical membership (e.g., insect, mammal) remained identifiable. To verify this, a separate group of 20 university students who did not participate in the main experiment rated how easily the image plus background sentence enabled inference of the correct semantic category (1 = very difficult; 5 = very easy). Materials were rated as effective in activating the target semantic category (*M* = 4.31, *SD* = 0.55), confirming that the combination of image and background sentence successfully conveyed categorical-level semantic information despite depicting a novel, imaginary referent. Importantly, the information conveyed was deliberately restricted to the superordinate category level, consistent with the level at which semantic radical effects have been shown to operate (Wang et al., 2026), and it did not specify any concrete semantic details of the novel word itself. This design does not render the paradigm intentional; participants were never informed of the need to memorize novel words, the post-test was withheld until after the learning phase, and all form–meaning connections were expected to be inferred incidentally through sentence reading (Hulstijn, 2003; Nation, 2022).

(3) Semantic category choice tasks

Immediately after presenting the activation materials, participants completed semantic category choice task 1, selecting the semantic category of the depicted and described concept from four options (see Figure 1, top-left, second panel). This task served as a manipulation check to verify that the activation materials successfully conveyed the intended superordinate category information; it did not involve any mapping between a novel word form and its meaning, and it therefore did not constitute explicit vocabulary instruction. After reading all nine sentences for a given novel word, participants completed semantic category choice task 2, which followed the same format as task 1 (see Figure 1, top-right) but assessed category inference based on sentence reading rather than activation materials. This task functioned as an online measure of semantic inference, parallel in purpose to the yes/no comprehension questions interspersed throughout the learning phase. Neither task required participants to retrieve or recognize the orthographic form of the novel words, nor did they direct attention to the vocabulary learning goal.

#### 2.3.2. Materials of Test Phase

To evaluate their incidental learning of orthographic and semantic knowledge after the learning phase, the participants completed two tasks, each comprising 16 trials presented in random order.

(1) Orthographic choice task.

Each trial presented four options: the target novel word; a visually similar pseudo-word differing by one or two strokes in one character; a pseudo-word sharing the same structure but with different radicals; and a completely unrelated pseudo-word. Participants indicated the item learned during reading by pressing the corresponding numeric key. Responses were scored as correct (1) or incorrect (0).

(2) Semantic category choice task.

Each trial presented four options: a specific entity most closely related to the meaning of the target novel word; another entity belonging to the same semantic category as the target novel word; the semantic category of a different novel word encountered during learning; and a concept not encountered during learning and not belonging to any of the eight categories. Participants were asked to select the option most semantically related to the target novel word. Scoring matched the orthographic task.

#### 2.3.3. Proficiency Assessments

Following the post-test, the participants completed three Chinese proficiency assessments to measure their orthographic awareness, vocabulary size, and semantic radical knowledge. These assessments included the following: (1) Real/Non-real Character Judgment (Chan & Chang, 2018). Participants completed an online character judgment test (LexTale-CH; available at https://gasparl.github.io/lextale/ (accessed on 1 July 2025)) indicating whether each item was a real Chinese character. The test comprised 90 items; the outcome was Corrected Accuracy, computed as h − 2 × f, where h is the number of correctly identified real characters (hits) and f is the number of incorrectly accepted nonce items (false alarms) (reliability = 0.88). (2) Real/Non-real Disyllabic Word Judgment (Wen et al., 2024). Participants completed a paper-based two-character word test judging whether each item was a real Chinese word. The test comprised 60 items; the outcome was a normalized Ghent score (Cronbach’s alpha = 0.96, see Wen et al. (2024)), computed as (*N* _yes to words_ − 2 × *N* _yes to nonwords_)/*N* _words_, yielding a fixed range of −100% to 100% and correcting for indiscriminate response tendencies regardless of item number. (3) Semantic Radical Knowledge Test. Based on Nguyen (2017), we developed a 40-item test for CFL learners. Each item presented one target radical in its typical position (e.g., “氵” on the left) and four small pictures depicting objects, actions, or body-related content. Participants selected the picture best matching the radical’s meaning. Responses were scored as the number of correct selections out of 40 (Cronbach’s alpha = 0.86).

### 2.4. Apparatus

Throughout the learning phase, participants’ eye movements were recorded using an Eyelink^®^ 1000 Plus eye-tracker system (SR Research, Ottawa, ON, Canada; sampling rate: 1000 Hz) as they read sentences from a computer monitor at a viewing distance of 75 cm. The learning contexts were presented in Song font size 18 (25 × 25 pixels), on an AOC 24B1XHM monitor with a resolution of 1024 × 768 pixels. Each character covered 0.75° of the horizontal visual angle. The post-test phase was programmed in E-Prime 2.0 and administered on an Asus FX50J laptop (ASUSTeK Computer Inc., Taipei, Taiwan). Screen resolution was set to 1024 × 768 pixels at 60 Hz. All stimuli were presented as black text on a white background.

### 2.5. Procedure

The experiment consisted of three parts: learning phase, test phase, and Chinese proficiency assessments. Participants were tested individually. After on-screen instructions and practice trials to ensure familiarity with the procedure, the formal learning phase began. For each novel word, participants first completed the semantic activation materials (self-paced via mouse click) and the semantic category choice task 1. A three-point horizontal calibration (mean error < 0.3°) was then administered immediately before the block of learning contexts. The nine learning contexts were presented one sentence per screen, with self-paced advancement. Three yes/no comprehension questions were randomly distributed across the nine learning exposures to ensure attentive reading. Upon completing the block, participants performed semantic category choice task 2. The blocks for different novel words were presented in random order. Participants were given one optional break during the learning session. The entire learning phase lasted approximately 30 to 50 min.

Immediately after the learning phase, the participants completed the tests. After practice, the orthographic choice task was administered first, followed by the semantic category choice task. Responses were recorded automatically; the test session lasted approximately 6 min.

Finally, participants completed the online character judgment (Chan & Chang, 2018), the paper-based disyllabic word judgment (Wen et al., 2024), and the paper-based semantic radical knowledge test. This part took approximately 15–20 min. At the end of the session, the experimenter debriefed participants regarding the nature of the pseudo-word materials and the purpose of the study.

### 2.6. Data Analysis

All analyses were conducted in R (version 4.2.2; R Core Team, 2022), a statistical computing environment. Linear mixed-effects models (LMMs) were fitted for continuous measures (time-based eye-movement measures, such as gaze duration, total fixation time); generalized LMMs (GLMMs) were used for binary measures (e.g., refixation probability, comprehension accuracy), using the lme4 package (Bates et al., 2015). For time-based eye-movement measures, values were log-transformed prior to modeling. Models with maximal random-effects structure were fitted where possible (Barr et al., 2013; Bates et al., 2015); reader group, semantic radical–category consistency, exposures (1–9, entered as a continuous predictor), and their interactions were entered as fixed effects; participants and items were treated as crossed random effects (Meteyard & Davies, 2020). Random structures were simplified when necessary to achieve convergence (Baayen et al., 2008).

We conducted analyses of eye-movement data for the target pseudoword in each learning sentence. The dependent measures included gaze duration (the cumulative duration of fixations on the target from its first fixation until a fixation on another word), refixation probability (the probability that the target area received more than one fixation during first-pass reading), regression path reading time (the sum of fixation durations from the first fixation on the target up to the first fixation to its right, excluding that rightward fixation, thereby capturing time associated with regressions and re-reading), and total fixation time (the cumulative duration of all fixations on the target word across the entire trial). Gaze duration is generally regarded as an early processing measure reflecting lexical-level processing of the target word, whereas regression path time indexes both lexical access and later sentence-integration processes involved in problem detection; total fixation time encompasses later processing, often associated with integration of the target word into a sentence context (Rayner et al., 1989).

For eye-tracking, invalid data were removed based on standard criteria: (1) trials with tracker loss or incomplete sentence reading; (2) trials with fewer than three fixations on single sentence; (3) individual fixations shorter than 80 ms or longer than 1200 ms (Rayner, 2009); and (4) data points deviating more than three standard deviations from the participant-by-condition cell mean for each time-based eye-movement measure. The overall trial exclusion rate was 12.90%.

## 3. Results

### 3.1. Reading Comprehension and Semantic Category Choice Accuracy During the Learning Phase

Comprehension accuracy across both reader groups and experimental conditions exceeded 89% (Table 3), indicating that the participants read the learning contexts attentively and understood their meaning. Mean accuracy for semantic category choice task 1 and task 2 exceeded 72%, suggesting that the activation materials effectively facilitated semantic category-level representations and that the readers were able to infer the novel word’s semantic category from the learning contexts.

GLMMs were fitted to test the effects of reader group, semantic radical–category consistency, and their interaction, with participants and items included as random effects. Exposure was not included as a predictor given that comprehension questions and category choice tasks were not designed to capture learning trajectories across exposures. Random structures were simplified stepwise from maximal models until convergence was achieved.

For reading comprehension, semantic radical–category consistency was not significant (*b* = 0.05, *SE* = 0.29, *z* = 0.18, *p* = 0.86), whereas the effect of reader group was significant (*b* = −0.63, *SE* = 0.30, *z* = −2.09, *p* = 0.04), with L1 readers outperforming CFL learners; the interaction was not significant (*b* = −0.24, *SE* = 0.30, *z* = −0.80, *p* = 0.43). For semantic category choice tasks 1 and 2, the effect of semantic radical–category consistency was not significant (*ps* > 0.50), whereas the effect of reader group was significant for both tasks (Task 1: *b* = −0.79, *SE* = 0.26, *z* = −3.06, *p* = 0.002; Task 2: *b* = −0.99, *SE* = 0.30, *z* = −3.36, *p* < 0.001), with L1 readers outperforming CFL learners in both tasks; the interactions were not significant (*ps* > 0.12). In summary, semantic radical–category consistency did not influence any of the three learning-phase measures, whereas L1 readers consistently outperformed CFL learners across all three tasks during learning.

### 3.2. Test Accuracy: Orthographic Choice and Semantic Category Choice

Regarding orthographic choice, one-sample t-tests confirmed that accuracy for both sets of novel words significantly exceeded chance level (25%, given four response options; *ts* > 10, *ps* < 0.001), indicating that the readers successfully acquired the orthographic representations of the novel words through exposure to the nine learning contexts, as evidenced by their performance on the orthographic choice task administered immediately after the learning phase. Table 4 presents the mean accuracy for both tasks across conditions and reader groups. GLMMs revealed that the effect of semantic radical–category consistency was not significant (*b* = 0.42, *SE* = 0.26, *z* = 1.61, *p* = 0.11). The effect of reader group was significant (*b* = −0.61, *SE* = 0.25, *z* = −2.42, *p* = 0.015), with L1 readers outperforming CFL learners; the interaction was not significant (*b* = −0.12, *SE* = 0.28, *z* = −0.45, *p* = 0.66).

Regarding semantic category choice, accuracy also significantly exceeded chance level (25%; *ts* > 8, *ps* < 0.001), indicating that readers acquired at least partial semantic representations of the novel words. GLMMs showed that the effect of semantic radical–category consistency was significant (*b* = −0.89, *SE* = 0.36, *z* = −2.47, *p* = 0.01), with consistent items yielding higher accuracy than inconsistent items. The effect of reader group was also significant (*b* = −1.32, *SE* = 0.22, *z* = −6.05, *p* < 0.001), with L1 readers outperforming CFL learners; the interaction was not significant (*b* = 0.45, *SE* = 0.29, *z* = 1.59, *p* = 0.11). Thus, semantic radical–category consistency facilitated semantic category learning, while L1 readers outperformed CFL learners in both orthographic and semantic choice tasks.

### 3.3. Eye-Movement Analyses

#### 3.3.1. Overall Learning Phase

The descriptive statistics for eye-movement measures across learning exposures are presented in Table 5 and the results of the LMMs are summarized in Table 6.

**Figure 2:**
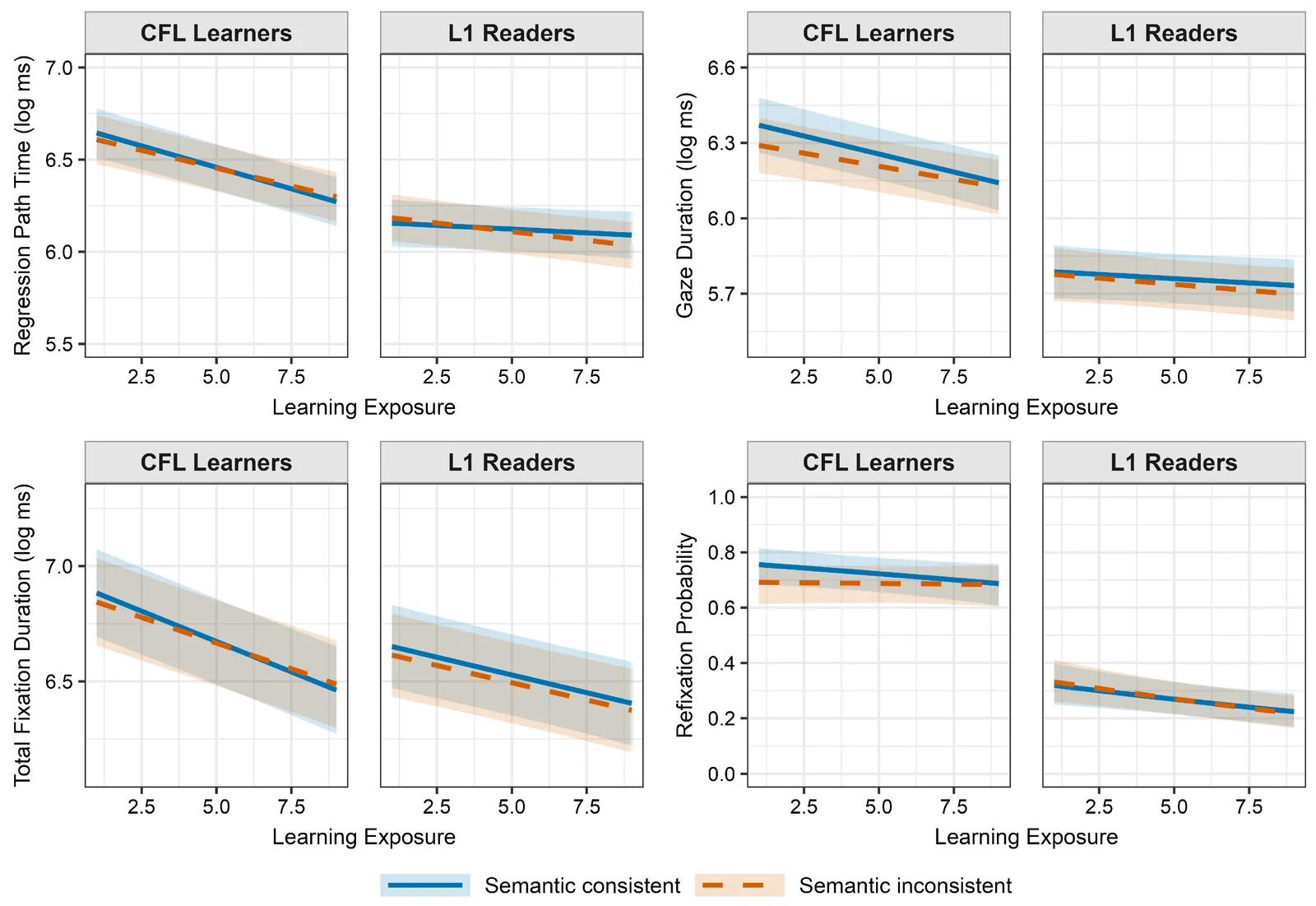
Comparison of the familiarity effect between the L1 readers and CFL learners across nine exposures.

Significant main effects of exposures were observed for gaze duration, refixation probability, regression path reading time, and total fixation time, indicating a clear downward trend in processing time as exposure increased. Similarly, significant main effects of reader group were found for gaze duration, refixation probability, and regression path reading time, with CFL learners showing longer fixation times and higher refixation probability than L1 readers. Furthermore, significant reader group × exposure interactions were observed across all four eye-movement measures (Figure 2). Over the course of the nine exposures, CFL learners exhibited stronger familiarity effects (i.e., steeper declines in processing time) in relation to gaze duration, regression path reading time, and total fixation time compared to L1 readers. In contrast, L1 readers exhibited stronger familiarity effects with regard to refixation probability.

**Figure 3:**
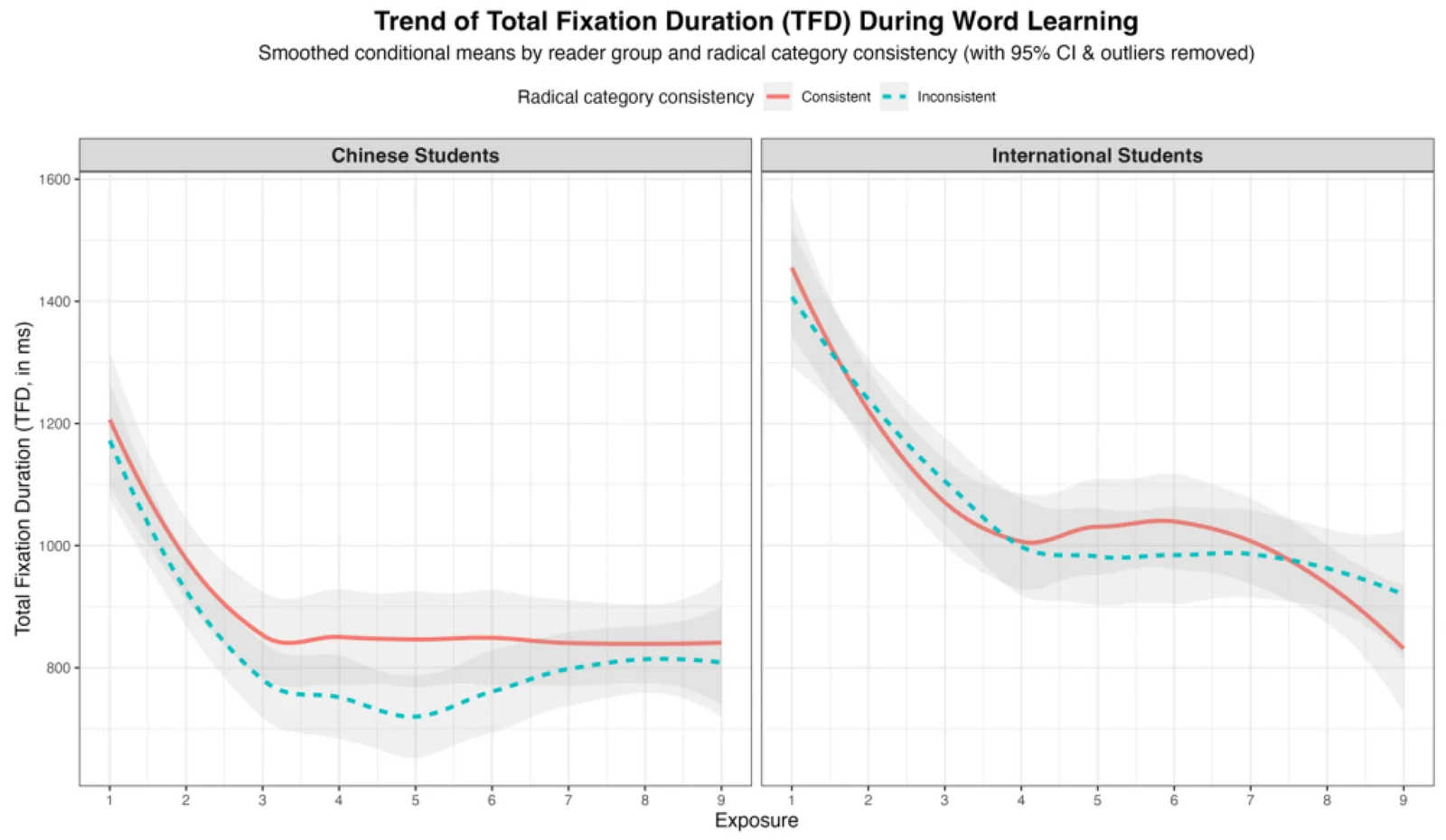
Smoothed trajectories and 95% confidence intervals for total fixation time on novel words during learning.

At the aggregate level, semantic radical–category consistency did not significantly affect novel-word processing during incidental vocabulary learning. A plausible explanation is that once the word processing approached a plateau phase (Elgort et al., 2018; Godfroid et al., 2018), differences in eye-tracking metrics between the two consistency conditions progressively diminished, thereby masking any effects of sublexical semantic decomposition that may have been present during the initial stages of learning. To verify the presence of a plateau, we fitted a spline regression (df = 3) to mean total fixation time across the nine exposures (Figure 3). As shown in the figure, the downward trend in total fixation time decelerated after the second exposure, marking the onset of the plateau phase.

Given that the plateau emerged after the second exposure, the first two exposures represent a critical window during which sublexical processing may exert its strongest influence on novel word recognition. To capture potential consistency effects that may have been obscured in the aggregate analysis, we conducted a focused examination of eye-tracking data from the first two exposures. The analytical approach for this subset was identical to that applied to the full learning phase.

#### 3.3.2. First Two Exposures

Analyses of the first two exposures (Table 7) showed significant exposure effects across all four eye-movement measures, confirming familiarity effects at the onset of novel word representation building. Significant reader group effects were found for gaze duration, re-fixation probability, and regression path reading time, with CFL learners exhibiting longer fixation times and higher refixation probabilities than L1 readers.

**Figure 4:**
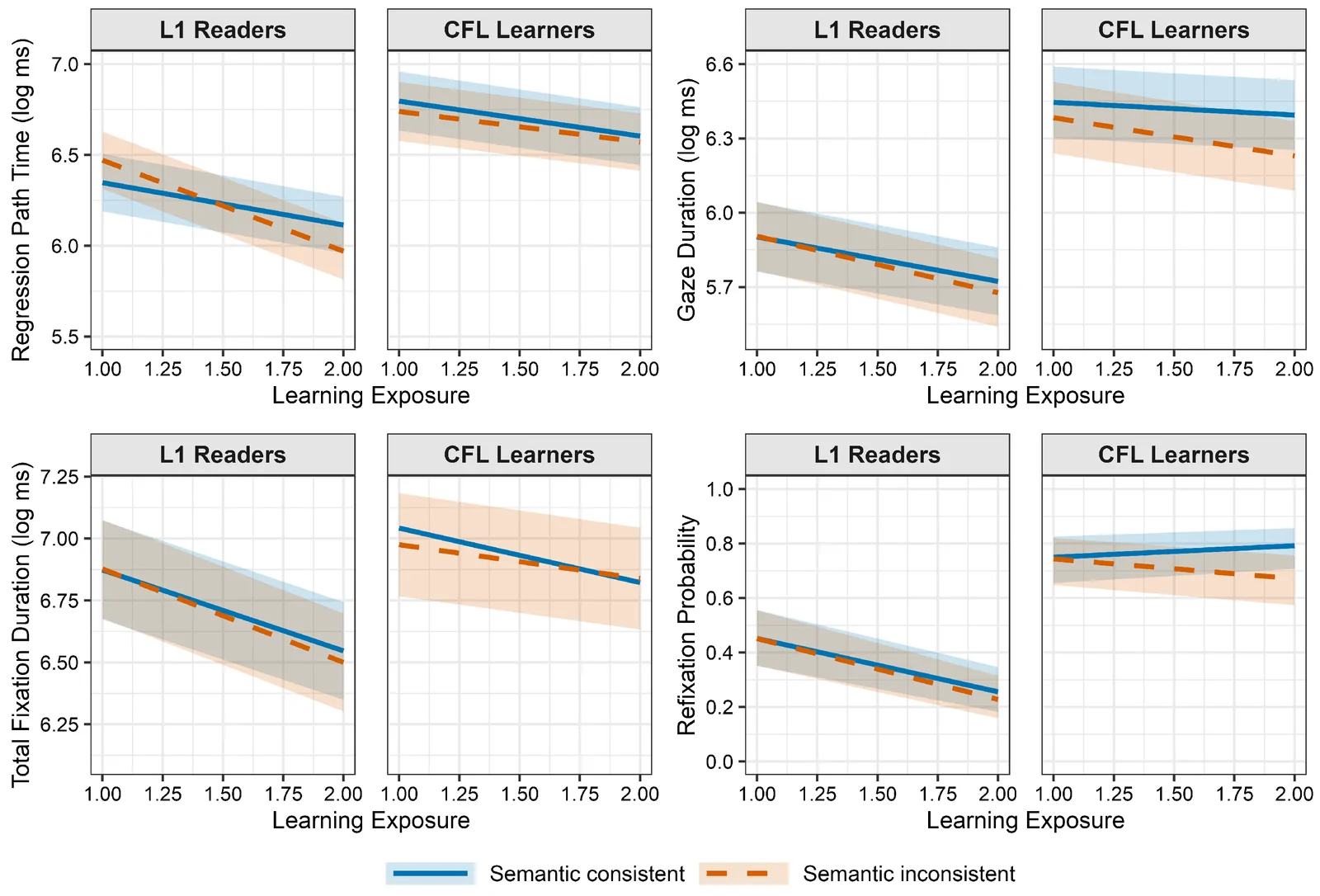
Comparison of the familiarity effect between the L1 readers and CFL learners during the first two exposures.

Crucially, significant reader group × exposure interactions were found for refixation probability, regression path reading time, and total fixation time. During the first two exposures, L1 readers exhibited stronger familiarity effects than CFL learners across these measures (Figure 4), suggesting that L1 readers shifted their processing strategies earlier in the initial stages of learning.

A significant three-way interaction among reader group × semantic radical–category consistency × exposure was observed for regression path reading time, indicating that the consistency effect varied by group and exposure. Simple-simple effect analyses revealed that for CFL learners, the radical consistency effect was not significant at either the first or second exposure (*ps* > 0.475). For L1 readers, however, the radical consistency effect changed across exposures; at the first exposure, inconsistent items elicited longer regression path reading times than consistent items, although the difference did not reach statistical significance (*b* = 0.122, *t* = 1.537, *p* = 0.128, *d* = 0.197); at the second exposure, inconsistent items elicited shorter regression path reading times than consistent items, showing a marginally significant trend (*b* = −0.143, *t* = −1.872, *p* = 0.065, *d* = −0.231). Together, these patterns suggest that L1 readers adjusted processing their processing strategies between the first and second exposures. Initially, they spontaneously extracted sublexical semantic cues from the novel word and detected conflicts between radical–category information and contextual semantics, allocating more processing resources to re-reading the preceding context; by the second exposure, they rapidly adapted, requiring less time for semantic integration of inconsistent items.

#### 3.3.3. Relationships Between Summed Total Reading Time and Learning Outcomes

To investigate the relationship between reading time investment during incidental word learning and subsequent learning outcomes, we examined associations between cumulative total reading times for novel words and accuracy on two post-learning tasks: semantic category judgment and orthographic judgment. Following Godfroid et al. (2018), we operationalized reading time investment as Summed Total Reading Time (STRT), calculated by progressively summing total fixation durations on the target novel word across exposures 1 through 9. For example, the STRT value at exposure 3 represents the cumulative sum of total fixation durations from the first three exposures to a given novel word. This cumulative measure captures the total amount of attention allocated to each word over the course of learning, offering a more complete picture of vocabulary acquisition than single-exposure or mean reading time measures.

Our dataset consisted of 944 participant-by-word observations (59 participants × 16 novel words). Each observation paired one participant’s cumulative reading time for a given novel word with their accuracy on the post-learning judgment task for that word. Prior to analysis, outliers exceeding ±3*SD* were removed separately for each of the nine STRT variables, resulting in 847 observations for both semantic category judgment and orthographic judgment tasks (exclusion rate = 10.3%). Log transformation was then applied to all STRT variables to reduce positive skew. Given that the outcome variables were binary (correct vs. incorrect) and the predictor variables were continuous (log-transformed STRT), point-biserial correlation coefficients (*r*_pb_) were computed to examine the linear association between cumulative reading time investment and learning performance.

(1) Overall correlations

Regarding the semantic category judgment task, the correlation analysis revealed a pattern of weak but consistently negative associations between log-transformed STRT and semantic category judgment accuracy across most learning phases. The correlation for exposure 1 was not significant (*r*_pb_ = −0.023, 95% CI [−0.090, 0.045], *p* = 0.512). However, from exposure 1–2 onward, negative correlations emerged consistently: exposure 1–2 (*r*_pb_ = −0.071, 95% CI [−0.138, −0.004], *p* = 0.039), exposure 1–3 (*r*_pb_ = −0.084, 95% CI [−0.150, −0.017], *p* = 0.015), exposure 1–4 (*r*_pb_ = −0.087, 95% CI [−0.154, −0.020], *p* = 0.011), exposure 1–5 (*r*_pb_ = −0.097, 95% CI [−0.163, −0.029], *p* = 0.005), exposure 1-6 (*r*_pb_ = −0.099, 95% CI [−0.165, −0.032], *p* = 0.004), exposure 1–7 (*r*_pb_ = −0.100, 95% CI [−0.167, −0.033], *p* = 0.003), exposure 1–8 (*r*_pb_ = −0.102, 95% CI [−0.168, −0.035], *p* = 0.003), and exposure 1–9 (*r*_pb_ = −0.098, 95% CI [−0.164, −0.030], *p* = 0.004). These negative correlations suggest that learners who invested more cumulative reading time tended to perform less accurately on the semantic category judgment task, perhaps reflecting processing difficulties or less efficient learning strategies.

In contrast to the semantic category judgment task, the orthographic judgment task showed a distinct trajectory. A significant positive correlation emerged at exposure 1 only (*r*_pb_ = 0.097, 95% CI [0.030, 0.163], *p* = 0.005), indicating that greater initial reading time investment was associated with better orthographic learning outcomes. However, beyond this initial encounter, the association weakened and was no longer significant: exposure 1–2 (*r*_pb_ = 0.064, 95% CI [−0.003, 0.131], *p* = 0.062), exposure 1–3 (*r*_pb_ = 0.043, 95% CI [−0.024, 0.110], *p* = 0.207), and exposure 1–4 through 1–9 (*r*_pb_s ranged from −0.001 to 0.023, all *p*s > 0.05). These findings suggest that early attentional investment in orthographic features may facilitate orthographic representation learning, whereas cumulative reading time beyond the initial exposure does not predict orthographic judgment accuracy.

(2) Group differences

To assess group differences in the relationship between reading time investment and learning outcomes, separate correlation analyses were conducted for L1 readers and CFL learners.

In the semantic category judgment task, for L1 readers, correlations between log-transformed STRT and semantic category judgment accuracy were consistently near zero and non-significant across all exposures (*r*_pb_s ranged from −0.007 to 0.054, all *p*s > 0.05). CFL learners showed a trend toward negative correlations, particularly at later learning exposures, although none reached statistical significance (*r*_pb_s ranged from −0.053 to 0.019, all *p*s > 0.05). Fisher’s Z tests revealed no significant between-group differences in correlation coefficients across any learning phase (*Z*s ranged from −0.25 to 1.64, all *p*s > 0.05), suggesting that the overall negative correlation observed in the combined sample was not driven by group differences.

In the orthographic judgment task, a more pronounced group difference emerged. L1 readers showed consistently positive correlations between log-transformed STRT and orthographic judgment accuracy across all learning phases: exposure 1 (*r*_pb_ = 0.140, *p* = 0.002), exposure 1–2 (*r*_pb_ = 0.124, *p* = 0.007), exposure 1–3 (*r*_pb_ = 0.134, *p* = 0.003), exposure 1–4 (*r*_pb_ = 0.120, *p* = 0.008), exposure 1–5 (*r*_pb_ = 0.109, *p* = 0.016), exposure 1–6 (*r*_pb_ = 0.119, *p* = 0.009), exposure 1–7 (*r*_pb_ = 0.104, *p* = 0.022), exposure 1–8 (*r*_pb_ = 0.109, *p* = 0.017), and exposuree 1–9 (*r*_pb_ = 0.100, *p* = 0.027). CFL learners, by contrast, exhibited consistently weaker and non-significant correlations (*r*_pb_s ranged from 0.022 to 0.085, all *p*s > 0.05), with the exception of a marginal trend at exposure 1 (*r*_pb_ = 0.085, *p* = 0.076). Fisher’s *Z* tests indicated that none of these group differences reached statistical significance (*Z*s ranged from 0.69 to 1.48, all *p*s > 0.05), although the consistently positive pattern for L1 readers and the near-zero pattern for CFL learners suggest a trend toward stronger orthographic learning benefits from investment in reading time among L1 readers.

(3) Effects of radical type

To examine whether semantic radical–category consistency moderated the relationship between reading time investment and semantic category judgment accuracy, correlations were calculated separately for words with semantically consistent radicals and words with semantically inconsistent radicals. For words with semantically consistent radicals, significant negative correlations emerged from exposure 1–2 onward: exposure 1–2 (*r*_pb_ = −0.117, *p* = 0.012), exposure 1–3 (*r*_pb_ = −0.119, *p* = 0.011), exposure 1–4 (*r*_pb_ = −0.115, *p* = 0.013), exposure 1–5 (*r*_pb_ = −0.122, *p* = 0.009), exposure 1–6 (*r*_pb_ = −0.128, *p* = 0.006), exposure 1–7 (*r*_pb_ = −0.131, *p* = 0.005), exposure 1–8 (*r*_pb_ = −0.132, *p* = 0.005), and exposure 1–9 (*r*_pb_ = −0.129, *p* = 0.005). For words with semantically inconsistent radicals, by comparison, all correlations were weak and non-significant (*r*_pb_s ranged from −0.037 to −0.003, *p*s > 0.05). Fisher’s Z tests revealed no significant differences between the two types of radical (*Z*s ranged from −0.64 to −1.67, *p*s > 0.05), although the pattern suggests a trend toward stronger negative associations for semantically consistent radicals.

## 4. Discussion

The present study examined incidental vocabulary learning of novel Chinese words among L1 readers and CFL learners. By manipulating the semantic radical–context consistency and combining eye tracking with offline vocabulary tests, we aimed to follow the time course of sublexical processing, investigating how it mapped onto learning outcomes. The results indicate successful acquisition of orthographic forms and partial meanings across groups, with L1 readers commanding a decisive advantage in performance. A more nuanced pattern emerged in the eye-movement data. While both groups showed a “familiarity effect”, with processing costs dropping across exposures, only L1 readers showed sensitivity to semantic radical consistency during initial exposure, when lexical representations were still being constructed online. Furthermore, the relationship between online processing time and learning outcomes was complex, varying by task and learner proficiency.

### 4.1. The Trajectory of Incidental Vocabulary Learning

Incidental learning seldom occurs as an instantaneous leap; it is typically an incremental process. Our findings align closely with the focus-enrichment model (Rieder, 2002) and with multi-exposure research in both L1 and L2 reading (Pellicer-Sánchez, 2016; Webb, 2019). Repeated exposure enabled both L1 readers and CFL learners to perform above chance on orthographic and semantic assessments. From a practical standpoint, exposure to informative sentences effectively fulfilled its functional role by providing readers with sufficient contextual cues over time to gradually establish form–meaning connections.

Across exposures, fixation times decreased, and regressions became less likely, in line with the “familiarity effect” reported in multi-exposure eye-tracking studies of incidental word learning, where the steepest reductions typically occur within the first few encounters (Pellicer-Sánchez, 2016; Elgort et al., 2018; Godfroid et al., 2018). What our data adds is a clear group-by-exposure interaction; CFL learners showed steeper declines in time-based measures (gaze duration, regression path reading time, and total fixation time), whereas L1 readers showed a stronger decline in refixation probability. At this stage, we treat these exposure-related slopes as descriptive signatures of how processing changes over repeated encounters. Separately, to contextualize the overall group difference in fixation-based measures (e.g., longer gaze duration in CFL learners), a narrower and better supported account attributes this difference to less secure orthographic knowledge in CFL learners compared with L1 readers (Shen & Ke, 2007). According to Mancheva et al. (2015), lower orthographic knowledge is reliably associated with longer fixation-based measures, including first-fixation duration and gaze duration. With that in mind, the longer gaze durations observed in our CFL learners are consistent with less secure orthographic knowledge than L1 readers, without requiring any stronger claim about sentence-level integration or rereading mechanisms (Mancheva et al., 2015). Still, the direction of the group difference aligns with the general claim in the linguistic proficiency hypothesis that eye-movement patterns are shaped primarily by lexical processing efficiency and post-lexical integration demands (Reichle et al., 2013; Mancheva et al., 2015). In addition, our spline analysis of total fixation time identified an inflection after the second exposure, after which reductions slowed markedly. This early change point is in line with the view that the earliest encounters are especially informative in incidental learning and therefore provides a principled rationale for focusing the semantic radical consistency analyses on the first two exposures (Borovsky et al., 2012; Share, 2004; Pellicer-Sánchez, 2016; Elgort et al., 2018; Godfroid et al., 2018; Zhang & Li, 2016).

### 4.2. The Role of Semantic Radicals in Online Processing

The present study examined whether readers exploit sublexical semantic cues during incidental word learning. The post-reading tests showed a reliable advantage of semantic radical–context consistency for semantic learning, consistent with accounts in which sublexical meaning cues help constrain form–meaning mappings when lexical representations are newly formed (Zhang & Li, 2016). However, the online record suggests that this effect did not surface as a uniform facilitation effect in eye movements across reader groups. Put differently, the sources of information that supported later semantic decisions were not necessarily expressed as immediate processing advantages during reading.

For L1 readers, the effect of semantic radical–context consistency was concentrated in the first two encounters, the phase in which familiarity effects tend to be steepest and eye-movement measures are most sensitive to changes in how novel words are processed (Pellicer-Sánchez, 2016; Elgort et al., 2018; Godfroid et al., 2018). At exposure 1, inconsistent items elicited longer regression-path reading times than consistent items, in the expected direction. Although this difference did not reach conventional significance, its direction was compatible with the interpretation that L1 readers registered the radical’s category cue and experienced increased difficulty with regard to integration when that cue conflicted with contextual meaning, prompting additional rereading. At exposure 2, the tendency reversed, with inconsistent items showing shorter regression-path reading times than consistent items. We treat this reversal as an exposure-dependent change in the influence of semantic radical information rather than as evidence for a specific mechanism. One possible but tentative interpretation is that after an initial encounter, L1 readers may reduce reliance on an unreliable sublexical cue for a given item, thereby limiting interference and enabling more efficient sentence-level integration on subsequent encounters. However, given that the difference associated with the first exposure did not reach conventional significance and that the reversal pattern is based on descriptive trends rather than a formally modelled interaction, this account should be treated as speculative and awaits confirmation from studies designed to test cue-weighting dynamics directly.

This exposure-bound modulation is broadly compatible with evidence that radical properties affect early-stage processing during incidental learning in L1 readers (He et al., 2026), while also underscoring that such effects may be most visible in measures tied to disruption and rereading (here, regression-path reading time) rather than in broader fixation-time indices.

CFL learners (HSK 4–5) showed no reliable semantic radical consistency effect in relation to their eye movements. This null pattern is theoretically informative because the literature suggests competing expectations. Offline work has indicated that learners can deploy radical knowledge strategically to support meaning inference (Zhang & Li, 2016), and the NCCP framework allows for the development of a semantically driven pathway in L2 Chinese character processing (Tong & Yip, 2015; Tong et al., 2016). In contrast, recent eye-tracking evidence suggests that intermediate learners may not spontaneously prioritize semantic radicals online and may allocate disproportionate attention to sublexical phonetic components (Wood et al., 2025). Our results align more closely with this latter profile; learners showed a semantic advantage of consistency in the post-test, yet their moment-by-moment reading did not reveal a detectable online sensitivity to the manipulation. A plausible interpretation is that knowledge of radicals is available for these learners but not yet automatized for real-time use during natural reading. According to the focus-enrichment view, incidental learning depends on how attention is distributed during reading (Rieder, 2002), not merely on what knowledge is present in principle. From the perspective of the linguistic proficiency hypothesis, limitations in lexical processing efficiency and post-lexical integration may further reduce the likelihood that sublexical semantic cues are deployed reliably under time constraints (Reichle et al., 2013). Under these conditions, learners may rely primarily on contextual information to sustain comprehension, with semantic radical information contributing more readily in tasks that encourage deliberate reflection (e.g., offline semantic judgments) than in continuous online processing.

### 4.3. Reading Time and Learning Outcomes: Effort vs. Difficulty

These results reveal a complex relationship between reading time investment in novel words and learning outcomes that varies by task type, learner group, and type of radical.

For orthographic learning, longer reading time at the very first encounter predicted higher accuracy. This pattern is consistent with the idea that early attention supports the establishment of a visual–orthographic trace (Smith, 1993). For semantic learning, the relationship went in the opposite direction; from Exposure 1–2 onward, longer cumulative STRT was associated with lower accuracy of semantic category judgment, especially for items with semantically consistent radicals. Rather than reflecting productive elaboration, extended fixation and rereading when reading continuous sentences are more plausibly indicators of repeated difficulties with integration. When integration remained slow across encounters, semantic representations appeared less likely to stabilize, a pattern consistent with earlier reports that longer gaze duration does not guarantee correct meaning inference (R. Williams & Morris, 2004). These findings highlight the multifaceted nature of novel word learning and suggest that the relationship between reading time investment and learning outcomes is not straightforward, but rather depends on the specific learning task, learner characteristics, and stimulus properties.

### 4.4. Limitations and Future Directions

Several limitations of the present study warrant consideration. First, the sample size, while adequate for the statistical models employed, could be expanded in future research to enhance the generalizability of the findings. Second, the CFL learners in this study were at an intermediate level (HSK 4–5). Future research should examine learners across a broader range of proficiency levels to map the developmental trajectory of radical awareness more comprehensively. It would be particularly valuable to determine the proficiency threshold at which CFL learners begin to show online sensitivity to semantic radicals comparable to that of L1 readers. Third, the present study did not strictly control for the L1 background of the CFL learners. Given that learners from character-based backgrounds (e.g., Japanese) may process Chinese orthography differently from those from alphabetic backgrounds (e.g., English), future studies should systematically investigate the effect of L1–L2 orthographic distance on the utilization of radical cues. Finally, while we examined immediate learning outcomes, longitudinal designs are needed to assess the retention of incidentally learned vocabulary and the long-term consolidation of form-meaning connections established during incidental reading. Finally, given that the present study employed a highly controlled laboratory paradigm with pseudo-characters and immediate post-tests, these findings should not be directly extrapolated to instructional contexts. Classroom-based replications using naturally occurring vocabulary, extended learning periods, and delayed retention measures are needed before any strong pedagogical recommendations regarding radical instruction can be responsibly made.

## 5. Conclusions

The present study provides evidence that incidental vocabulary learning of novel Chinese words engages partially distinct cognitive mechanisms in L1 readers and CFL learners. Whereas L1 readers demonstrated online sensitivity to semantic radical–context consistency during initial exposure, CFL learners showed a consistency advantage only in offline assessments, suggesting that radical knowledge is available but not yet automatically deployed during real-time reading at intermediate proficiency levels. Reading time investment further proved to be a task-specific predictor of learning, being beneficial for orthographic learning at first exposure, but negatively associated with semantic accuracy over cumulative exposures. Taken together, these findings underscore the complexity of Chinese vocabulary acquisition and point to the necessity of targeted instructional interventions to help CFL learners attend to the internal semantic structure of Chinese characters during reading.

## Figures and Tables

**Table 1:** Descriptive statistics and group comparisons for L1 readers and CFL learners on three measures.

	LEXTALE Score		Vocabulary Score (%)		Radical Awareness Score	
	L1 Readers	CFL Learners	L1 Readers	CFL Learners	L1 Readers	CFL Learners
M	43.00	7.00	1.00	0.175	35.50	28.75
(Q_L_,Q_U_)	(41.00, 46.00)	(2.00, 14.00)	(0.95, 1.00)	(0.05, 0.25)	(34.50, 36.50)	(25.75, 32.00)
*M* ± *SD*	43.00 ± 4.40	8.85 ± 9.86	0.97 ± 0.05	0.15 ± 0.25	35.39 ± 1.22	28.61 ± 4.66
*Z*	−6.474		−6.660		−5.633	
*p*	<0.001		<0.001		<0.001	
Effect size *r*	−0.85		−0.87		−0.73	

**Table 2:** Illustration of example stimuli in the learning phase.

Exposure	Sentences
1	生长30天后那些白色 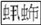 就能学会飞行。
2	农民寻找 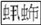 吐出的材料制作衣服。
3	小鸟会把 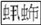 当成自己的食物。
4	花园里有许多 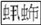 在阳光下闪闪发亮。
5	晒干后的黑色 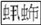 可以用来制作药物。
6	农民利用小 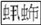 抓那些吃菜叶的虫子。

Note: Target pseudo-words are enclosed in boxes. The boxes did not appear when the sentences were presented to participants. The sentences translate respectively as: (1) “After 30 days of growth, those white 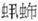 will be able to learn to fly.” (2) “The farmer looks for material spit out by 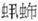 to make clothes.” (3) “Birds regard the 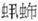 as their food.” (4) “There are many 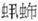 in the garden shining in the sunlight.” (5) “Dried black 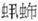 can be used to make medicine.” (6) “The farmer uses small 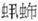 to catch insects that eat vegetable leaves.” The semantic radical “虫” refers to insect, and its meaning was closely related to the learning contexts, which imparted meaning to the entire novel word as a kind of worm, in accordance with the semantic radical’s consistent condition. The original Chinese punctuation 「。」 has been retained to accurately represent the experimental stimuli.

**Table 3:** Mean accuracy of comprehension questions and semantic category choice tasks in the two conditions for participants.

Task in Learning Phase	Consistent Semantic Radical Condition		Inconsistent Semantic Radical Condition	
	L1 Readers	CFL Learners	L1 Readers	CFL Learners
Reading comprehension	0.95 (0.22)	0.91 (0.29)	0.95 (0.21)	0.89 (0.31)
Semantic category choice task 1	0.84 (0.37)	0.73 (0.44)	0.90 (0.30)	0.72 (0.44)
Semantic category choice task 2	0.89 (0.31)	0.77 (0.42)	0.90 (0.30)	0.73 (0.44)

Note: Standard deviations are shown in parentheses.

**Table 4:** Mean accuracy of orthographic choice task and semantic category judgment in the two conditions for participants.

Task in Testing Phase	Consistent Semantic Radical Condition		Inconsistent Semantic Radical Condition	
	L1 Readers	CFL Learners	L1 Readers	CFL Learners
Orthographic choice	0.61 (0.49)	0.47 (0.50)	0.70 (0.46)	0.54 (0.50)
Semantic category judgment	0.77 (0.42)	0.50 (0.50)	0.61 (0.49)	0.41 (0.49)

Note: Standard deviations are shown in parentheses.

**Table 5:** Mean reading times (ms) and refixation probability (%) for novel words across learning exposures by reader group and semantic radical consistency.

Exposure	Refixation Probability				Regression Path Reading Time			
	L1 Readers		CFL Learners		L1 Readers		CFL Learners	
	Consistent Radical	Inconsistent Radical	Consistent Radical	Inconsistent Radical	Consistent Radical	Inconsistent Radical	Consistent Radical	Inconsistent Radical
1	45.9% (49.9%)	45.2% (49.9%)	74.4% (43.7%)	74.3% (43.8%)	760 (661)	880 (795)	1196 (1196)	1081 (848)
2	26.7% (44.3%)	25.1% (43.5%)	77.9% (41.6%)	66.7% (47.3%)	604 (588)	507 (431)	891 (681)	936 (1097)
3	28.2% (45.1%)	29.2% (45.6%)	73.8% (44.1%)	71.3% (45.3%)	522 (443)	547 (501)	861 (754)	822 (669)
4	24.8% (43.3%)	28.4% (45.2%)	71.2% (45.4%)	69.7% (46.1%)	615 (569)	574 (577)	788 (652)	823 (539)
5	21.6% (41.2%)	23.8% (42.7%)	72.0% (45.0%)	72.1% (45.0%)	562 (580)	558 (610)	797 (856)	808 (850)
6	27.6% (44.8%)	28.4% (45.2%)	74.3% (43.8%)	62.7% (48.5%)	497 (569)	524 (504)	811 (821)	811 (752)
7	28.6% (45.3%)	28.1% (45.1%)	68.7% (46.5%)	66.8% (47.2%)	650 (648)	592 (574)	737 (589)	722 (638)
8	24.0% (42.8%)	23.9% (42.7%)	72.4% (44.8%)	69.0% (46.4%)	624 (739)	578 (538)	796 (773)	690 (486)
9	29.4% (45.7%)	25.4% (43.6%)	68.9% (46.4%)	74.1% (43.9%)	660 (654)	608 (643)	633 (466)	770 (683)
**Exposure**	**Gaze Duration**				**Total Fixation Time**			
	**L1 Readers**		**CFL Learners**		**L1 Readers**		**CFL Learners**	
	**Consistent** **Radical**	**Inconsistent** **Radical**	**Consistent** **Radical**	**Inconsistent** **Radical**	**Consistent** **Radical**	**Inconsistent** **Radical**	**Consistent** **Radical**	**Inconsistent** **Radical**
1	460 (291)	477 (352)	795 (539)	763 (530)	1254 (1240)	1212 (933)	1470 (1200)	1395 (1002)
2	361 (241)	357 (260)	690 (380)	615 (420)	889 (725)	849 (744)	1191 (906)	1268 (1131)
3	366 (261)	352 (213)	618 (359)	641 (435)	838 (711)	768 (618)	1080 (759)	1080 (845)
4	360 (236)	354 (232)	617 (346)	605 (324)	887 (855)	771 (599)	1025 (739)	1058 (785)
5	353 (245)	326 (192)	602 (350)	564 (312)	813 (664)	718 (647)	976 (781)	901 (698)
6	376 (267)	358 (233)	635 (400)	566 (344)	864 (833)	724 (580)	1121 (994)	1065 (802)
7	363 (242)	365 (250)	581 (375)	558 (303)	856 (788)	878 (854)	940 (717)	946 (707)
8	363 (263)	358 (249)	601 (362)	553 (314)	804 (766)	782 (691)	963 (734)	979 (893)
9	396 (279)	363 (253)	520 (229)	550 (287)	854 (706)	814 (694)	827 (600)	917 (685)

Note: Standard deviations are shown in parentheses.

**Table 6:** Summary of linear mixed-effect model (LMM) analyses for all eye-movement measures.

Measure		*b*	95% CI	*SE*	*t/z*
Gaze duration					
	Intercept	5.990	[5.924, 6.056]	0.033	181.70
	**Group**	**0.481**	**[0.33, 0.631]**	**0.075**	**6.41**
	Consistency of semantic radical	−0.035	[−0.079, 0.008]	0.020	−1.73
	**Exposure**	**−0.043**	**[−0.054, −0.031]**	**0.006**	**−7.07**
	Learned radical knowledge	−0.036	[−0.11, 0.039]	0.037	−0.95
	Group × Radical	-0.026	[-0.08, 0.027]	0.027	-0.98
	**Group × Exposure**	**−0.042**	**[−0.066, −0.019]**	**0.012**	**−3.52**
	Radical × Exposure	0.006	[-0.018, 0.03]	0.012	0.51
	Group × Radical × Exposure	0.028	[-0.019, 0.076]	0.024	1.17
Refixation probability					
	Intercept	−0.063	[−0.256, 0.129]	0.098	−0.65
	**Group**	**1.874**	**[1.437, 2.311]**	**0.223**	**8.41**
	Consistency of semantic radical	−0.079	[−0.219, 0.06]	0.071	−1.11
	**Exposure**	**−0.118**	**[−0.174, −0.062]**	**0.029**	**−4.14**
	Learned radical knowledge	−0.156	[−0.373, 0.061]	0.111	−1.41
	Group × Radical	−0.177	[−0.39, 0.037]	0.109	−1.62
	**Group × Exposure**	**0.121**	**[0.007, 0.235]**	**0.058**	**2.08**
	Radical × Exposure	0.034	[−0.074, 0.142]	0.055	0.61
	Group × Radical × Exposure	0.128	[−0.088, 0.344]	0.110	1.16
Regression path reading time					
	Intercept	6.286	[6.205, 6.367]	0.040	155.49
	**Group**	**0.338**	**[0.157, 0.518]**	**0.090**	**3.74**
	Consistency of semantic radical	−0.010	[−0.073, 0.053]	0.030	−0.34
	**Exposure**	**−0.072**	**[−0.089, −0.054]**	**0.009**	**−8.29**
	Learned radical knowledge	−0.065	[−0.155, 0.025]	0.045	−1.44
	Group × Radical	0.012	[−0.058, 0.081]	0.035	0.34
	**Group × Exposure**	**−0.076**	**[−0.111, −0.042]**	**0.017**	**−4.42**
	Radical × Exposure	−0.004	[−0.032, 0.025]	0.015	−0.25
	Group × Radical × Exposure	0.048	[−0.009, 0.105]	0.029	1.65
Total fixation time					
	Intercept	6.590	[6.47, 6.71]	0.060	109.79
	Group	0.157	[−0.113, 0.427]	0.135	1.16
	Consistency of semantic radical	−0.020	[−0.103, 0.062]	0.039	−0.52
	**Exposure**	**−0.102**	**[−0.115, −0.089]**	**0.007**	**−15.15**
	Learned radical knowledge	−0.106	[−0.24, 0.027]	0.067	−1.59
	Group × Radical	0.029	[−0.058, 0.116]	0.043	0.67
	**Group × Exposure**	**−0.047**	**[−0.074, −0.021]**	**0.013**	**−3.51**
	Radical × Exposure	0.011	[−0.016, 0.037]	0.013	0.80
	Group × Radical × Exposure	0.020	[−0.033, 0.073]	0.027	0.75

Note: Values in bold indicate statistically significant effects.

**Table 7:** Summary of LMM analyses for all eye-movement measures in the first two exposures.

Measure		*b*	95% CI	*SE*	*t*/*z*
Gaze duration					
	Intercept	6.082	[6.001, 6.164]	0.041	149.92
	**Group**	**0.560**	**[0.376, 0.744]**	**0.092**	**6.10**
	Consistency of semantic radical	−0.068	[−0.144, 0.008]	0.036	−1.91
	**Exposure**	**−0.076**	**[−0.108, −0.045]**	**0.016**	**−4.81**
	Learned radical knowledge	0.008	[−0.084, 0.099]	0.046	0.17
	Group × Radical	−0.093	[−0.208, 0.022]	0.058	−1.59
	Group × Exposure	0.050	[−0.014, 0.113]	0.032	1.56
	Radical × Exposure	−0.038	[−0.095, 0.019]	0.029	−1.30
	Group × Radical × Exposure	−0.027	[−0.141, 0.088]	0.058	−0.45
Refixation probability					
	Intercept	0.185	[−0.032, 0.402]	0.111	1.67
	**Group**	**1.724**	**[1.229, 2.22]**	**0.253**	**6.82**
	Consistency of semantic radical	−0.216	[−0.461, 0.029]	0.125	−1.73
	**Exposure**	**-0.249**	**[−0.377, −0.122]**	**0.065**	**−3.83**
	Learned radical knowledge	−0.095	[−0.336, 0.146]	0.123	−0.77
	Group × Radical	−0.245	[−0.713, 0.224]	0.239	−1.02
	**Group × Exposure**	**0.508**	**[0.247, 0.77]**	**0.133**	**3.81**
	Radical × Exposure	−0.200	[−0.435, 0.034]	0.120	−1.67
	Group × Radical × Exposure	−0.228	[−0.697, 0.24]	0.239	−0.96
Regression path reading time					
	Intercept	6.442	[6.349, 6.536]	0.047	138.39
	**Group**	**0.445**	**[0.245, 0.646]**	**0.100**	**4.45**
	Consistency of semantic radical	−0.031	[−0.139, 0.076]	0.051	−0.61
	**Exposure**	**−0.137**	**[−0.172, −0.102]**	**0.017**	**−7.85**
	Learned radical knowledge	−0.027	[−0.126, 0.072]	0.049	−0.55
	Group × Radical	−0.024	[−0.169, 0.12]	0.072	−0.34
	**Group × Exposure**	**0.094**	**[0.025, 0.164]**	**0.035**	**2.71**
	Radical × Exposure	−0.060	[−0.122, 0.003]	0.032	−1.88
	**Group × Radical × Exposure**	**0.144**	**[0.019, 0.27]**	**0.064**	**2.26**
Total fixation time					
	Intercept	6.809	[6.682, 6.936]	0.063	107.29
	Group	0.219	[−0.069, 0.508]	0.144	1.52
	Consistency of semantic radical	−0.023	[−0.116, 0.071]	0.044	−0.52
	**Exposure**	**−0.133**	**[−0.161, −0.105]**	**0.014**	**−9.33**
	Learned radical knowledge	−0.077	[−0.221, 0.067]	0.072	−1.07
	Group × Radical	−0.006	[−0.132, 0.12]	0.063	−0.10
	**Group × Exposure**	**0.086**	**[0.031, 0.142]**	**0.028**	**3.04**
	Radical × Exposure	0.009	[−0.047, 0.065]	0.028	0.32
	Group × Radical × Exposure	0.066	[−0.046, 0.177]	0.057	1.15

Note: Values in bold indicate statistically significant effects.

## Data Availability

The data presented in this study are available on request from the corresponding author due to privacy and ethical restrictions protecting participant confidentiality.

## Supplementary Materials

The following supporting information can be downloaded at: https://www.mdpi.com/article/10.3390/bs16081317/s1, The supplementary PDF file contains the 144 experimental learning sentences used in this study.

## Abbreviations

The following abbreviations are used in this manuscript:

CFL	Chinese as a foreign language
LMMs	Linear mixed-effects models

## Appendix A

**Table A1:** Novel words.

Number	Semantic Category	Novel Words with Consistent Semantic Radical	Pronunciation of Semantic Radical	Novel Words with Inconsistent Semantic Radical	Pronunciation of Semantic Radical
1	Soil		土, tǔ		车, chē
2	Insect		虫, chóng		饣, shí
3	Food		饣, shí		土, tǔ
4	Vehicle		车, chē		虫, chóng
5	Plant		木, mù		女, nǚ
6	Metal products		钅, jīn		木, mù
7	Female occupation		女, nǚ		犭, quǎn
8	Mammal		犭, quǎn		钅, jīn
