# Supplementary material for: Semantic Radical Consistency in Incidental Chinese Vocabulary Learning: A Comparison of Native Readers and Chinese as a Foreign Language (CFL) Learners
Source: Behav Sci (Basel). 2026 Aug 3;16(8):1317. doi: 10.3390/bs16081317 (PMC13509858; doi:10.3390/bs16081317)
Supplement: Supplementary file 1 [file behavsci-16-01317-s001.zip › behavsci-4308180-supplementary-contain English version.pdf]

Learning context (Chinese)	Learning context (English)	semantic type
这座山上**占三分之二，非常适合种茶树。	** covers two-thirds of this mountain, making it very suitable for growing tea trees.	soil
科学家研究**中的物质如何使污水变干净。	Scientists are studying how substances in ** can purify sewage.	soil
这里的农民把**倒在土里，玉米会长得更好。	Farmers here pour ** into the soil, and the corn will grow better.	soil
花园里的路是用**和水加固的小路。	The paths in the garden are small trails reinforced with ** and water.	soil
手工店的人在**里加点水用来做成漂亮的瓶子。	People at the handicraft shop add some water to ** to make beautiful bottles.	soil
孩子们喜欢在**上坐着玩游戏。	Children like to sit on ** and play games.	soil
专家在十几米**底下发现了古代工具。	Experts discovered ancient tools more than ten meters below the **.	soil
古代的建筑工人会用**盖房子。	Ancient construction workers would use ** to build houses.	soil
下雨过后**会流出清水，当地人用它制作酒。	After rain, clear water will flow out from **, which locals use to make wine.	soil
山上这一片**种玉米特别好，秋天能收很多。	This patch of ** on the mountain is particularly good for growing corn, and a lot can be harvested	soil
工人用**种一种少见的花。	Workers use ** to grow a rare type of flower.	soil
下雨后，孩子们把湿**做成各种小动物的形状。	After rain, children shape wet ** into various small animals.	soil
人走在**上会留下明显的印子。	People walking on ** will leave obvious marks.	soil
刮大风时如果有**飞进眼睛一定要马上用水冲洗干净。	If ** flies into your eyes during strong winds, you must immediately rinse them with water.	soil
建筑工人往**里加海水用来修路。	Construction workers add seawater to ** for road construction.	soil
海边的大量**能吸收很多水分，人们会用它来种花。	The large amount of ** by the seaside can absorb a lot of moisture, and people use it to grow flowe	soil
弟弟在海边跑了一圈回家发现鞋子里有很多**根本倒不干净	After running a lap at the beach, my younger brother came home and found his shoes full of ** tha	soil
老师曾经说黑色**能帮助植物生长出根。	The teacher once said that black ** can help plants develop roots.	soil
生长30天后那些白色**就能学会飞行。	After 30 days of growth, those white ** can learn to fly.	insects
农民寻找**吐出的材料制作衣服。	Farmers search for materials spit out by ** to make clothes.	insects
小鸟会把**当成自己的食物。	Small birds will treat ** as their food.	insects
花园里有许多**在阳光下闪闪发亮。	There are many ** in the garden sparkling in the sunlight.	insects
晒干后的黑色**可以用来制作药物。	Dried black ** can be used to make medicine.	insects
农民利用小**抓那些吃菜叶的虫子。	Farmers use small ** to catch insects that eat vegetable leaves.	insects
下大雨之前**会爬到更高的地方。	Before heavy rain, ** will climb to higher places.	insects
科学家发现**在夏天会咬人。	Scientists have discovered that ** will bite people in summer.	insects
学生在科学课上观看**生长的过程。	Students watch the growth process of ** in science class.	insects
弟弟发现**在搬小面包，看了十分钟。	My younger brother found ** carrying small pieces of bread and watched for ten minutes.	insects
爷爷看到一群**排队搬家就说要下雨了。	When grandpa sees a group of ** lining up to move, he says it's going to rain.	insects
妈妈擦桌子时发现**爬进蔬菜汤里了。	Mom found ** had crawled into the vegetable soup when wiping the table.	insects
老师让我们观察**怎么运食物。	The teacher asked us to observe how ** transport food.	insects
令我们吃惊的是**可以搬动比自己重很多的东西。	To our surprise, ** can carry things much heavier than themselves.	insects
故事书里**把树叶当成小船来过河。	In the storybook, ** use leaves as small boats to cross the river.	insects

孩子们用树叶帮**挡住雨水。	Children use leaves to help ** block the rain.	insects
大自然中某些**能在天上飞。	In nature, some ** can fly in the sky.	insects
爸爸把能杀死虫子的药倒在门口防止**爬进房间。	Dad poured insect-killing medicine at the doorway to prevent ** from crawling into the room.	insects
早餐店有**做成的包子很好吃。	The breakfast shop has buns made with ** that are very delicious.	food
妈妈做饭会加一点点**和蔬菜，特别好吃。	Mom adds a little bit of ** and vegetables when cooking, which is especially delicious.	food
没放进冰箱的那些**在夏天很快就坏了。	Those ** that weren't put in the refrigerator spoiled quickly in summer.	food
中秋节那天很多小店摆出用**做的好看的月饼。	On Mid-Autumn Festival, many small shops displayed beautiful mooncakes made with **.	food
加一点点**可以让蛋糕有独特的味道。	Adding a little bit of ** can give the cake a unique flavor.	food
气温低的时候**可以放三个月。	When the temperature is low, ** can be kept for three months.	food
胖的人也可以吃那些用**制作的食物。	Fat people can also eat food made with **.	food
小明的爸爸喜欢把**和肉放在汤里一起吃。	Xiao Ming's father likes to put ** and meat in soup to eat together.	food
食堂阿姨做饭的时候发现很多**都用完了。	The cafeteria auntie found that a lot of ** had been used up when cooking.	food
古代的中国人会在**里加一种特别的叶子，制作成类似咖啡的东	Ancient Chinese people would add a special kind of leaf to ** to make something similar to coffee.	food
在0-6℃的水里加**会形成一层绿色的东西，像酸奶一样。	Adding ** to water at 0-6℃ will form a layer of green stuff, like yogurt.	food
小朋友们看到**上面的那一层巧克力都觉得很神奇。	The children all find the layer of chocolate on top of ** very magical.	food
一个小朋友喝了一杯**后说就像在喝有气的果汁。	A child said after drinking a cup of ** that it was like drinking carbonated fruit juice.	food
弟弟不小心把**弄到作业本上，急得哭起来。	My younger brother accidentally got ** on his homework and cried anxiously.	food
如果大家都用玻璃杯喝**就能更好地保护环境。	If everyone uses glass cups to drink **, it can better protect the environment.	food
朋友喜欢在**里面加牛奶，这样更好喝。	My friend likes to add milk to **, which makes it taste better.	food
他们店这杯**卖十五元，太贵了。	This cup of ** at their shop sells for fifteen yuan, too expensive.	food
熊猫图案的杯子是这家**店的特色。	The panda-patterned cup is the signature feature of this ** shop.	food
自行车的速度比**慢许多。	The speed of a bicycle is much slower than **.	vehicles
这辆绿色**能自动开门关门并保证乘客安全。	This green ** can automatically open and close doors and ensure passenger safety.	vehicles
修理师傅在更换**出问题的设备。	The repairman is replacing the malfunctioning equipment of **.	vehicles
早晨上班时间**每十分钟发一班，非常方便。	During morning rush hour, ** departs every ten minutes, which is very convenient.	vehicles
想要进入**必须要经过安全检查。	To enter **, you must go through a security check.	vehicles
孩子们在玩**形状的玩具。	Children are playing with **-shaped toys.	vehicles
小男孩在**上大叫吵到了其他人，妈妈批评了他。	A little boy shouted loudly on ** and disturbed other people, so his mother criticized him.	vehicles
6岁以下的儿童坐**全部免费。	Children under 6 years old can ride ** for free.	vehicles
放假的时候有很多人坐**去旅行。	During holidays, many people take ** to travel.	vehicles
去海边旅游要坐大**才能到达。	To travel to the seaside, you need to take a large ** to get there.	vehicles
天空中的那个白色长线是**开过后留下的。	The white long line in the sky is left behind after ** passes by.	vehicles
老师给小学生说**能在天上飞行的原因。	The teacher explains to elementary school students the reason why ** can fly in the sky.	vehicles
燃烧特殊的燃料是**高速飞行的原因	Burning special fuel is the reason why ** flies at high speed.	vehicles

有经验的司机才敢在打雷下雨的天气给**补充燃料。	Only experienced drivers dare to refuel ** in thunderstorm weather.	vehicles
科学家正在研究让**同时使用两种燃料的方法。	Scientists are researching methods to make ** use two types of fuel simultaneously.	vehicles
工人们把很大的东西直接放进**运往国外。	Workers put very large things directly into ** to transport them abroad.	vehicles
计划旅游的人会买**商务座位的票。	People planning to travel will buy tickets for ** business class seats.	vehicles
人们需要提前三小时办理**乘坐手续。	People need to check in for ** three hours in advance.	vehicles
古书中记载了人们用**叶子制作有颜色的纸。	Ancient books recorded that people used ** leaves to make colored paper.	plants
我听到那片**林传来的鸟叫能感到平静。	I can feel calm when I hear the bird calls coming from that ** forest.	plants
只有超过100岁的老人见过**果实真正的样子。	Only elderly people over 100 years old have seen what ** fruit really looks like.	plants
最新论文发现**适应环境的能力很强。	The latest research paper found that ** has a strong ability to adapt to the environment.	plants
小朋友们在书上看到红色**觉得很特别。	Children find red ** very special when they see it in books.	plants
博物馆里有大型**化石参观。	There are large ** fossils to visit in the museum.	plants
当地人说如果想实现愿望可以在**上挂红色的布。	Locals say if you want to make a wish come true, you can hang red cloth on **.	plants
科学家通过**化石研究古代气候。	Scientists study ancient climate through ** fossils.	plants
中国古代的人认为**会带来幸运。	Ancient Chinese people believed that ** would bring good luck.	plants
在古代森林中**曾是最常见的。	In ancient forests, ** was once the most common.	plants
由于气候的变化**渐渐从地球上消失。	Due to climate change, ** gradually disappeared from the Earth.	plants
许多生物曾把**作为食物来源。	Many organisms once used ** as a food source.	plants
在古时候**是绿色的并且有刺。	In ancient times, ** was green and had thorns.	plants
中国古代的山水画可以证明**曾经存在过。	Ancient Chinese landscape paintings can prove that ** once existed.	plants
科学家通过研究**化石解释了地球的历史。	Scientists explained the history of the Earth by studying ** fossils.	plants
环境保护部门用**作为例子说明保护环境的重要作用。	Environmental protection departments use ** as an example to illustrate the important role of prote	plants
森林里的动物经常会被**刺伤，流很多血。	Animals in the forest are often injured by **, bleeding a lot.	plants
古代的人用**治疗感冒。	Ancient people used ** to treat colds.	plants
古代的人把**安装在门上象征财富。		metal products
科学家在大山的深处发现**发出蓝色的光。	Ancient people installed ** on doors to symbolize wealth.	metal products
这种产品是用**制造的，有长期保存的价值。	Scientists discovered ** emitting blue light in the depths of the mountains.	metal products
古代人用火把**烧成不同的形状。	This product is made with **, and has long-term preservation value.	metal products
在博物馆里我们看到了古代人用**制作的武器。	Ancient people used fire to burn ** into different shapes.	metal products
学者研究发现**燃烧能放出能量。	In the museum, we saw weapons made by ancient people with **.	metal products
这种镜子表面是用**制成的，看起来非常清楚。	Scholars' research found that burning ** can release energy.	metal products
这家手机维修店有很多用**制作的材料。	The surface of this mirror is made with **, and it looks very clear.	metal products
他们想要用**制作一个闪闪的杯子。	This mobile phone repair shop has many materials made with **.	metal products
研究生在报告中讨论**中的神秘物质。	They want to use ** to make a sparkling cup.	metal products
在公园里用**烤肉熟得很快。	Graduate students discuss the mysterious substance in ** in their report.	metal products

我们需要准备几个**方便明天活动时使用。	In the park, meat roasted with ** cooks very quickly.	metal products
我们学校买了一批**用于实验室。	We need to prepare several ** for convenient use in tomorrow's activity.	metal products
他们设计了一种新型**可以自由改变形状。	Our school bought a batch of ** for the laboratory.	metal products
工厂生产这种**已经有五年的时间了。	They designed a new type of ** that can freely change shape.	metal products
妈妈喜欢用**加热牛奶。	The factory has been producing this ** for five years.	metal products
他写了一篇文章介绍**在厨房里的用途。	Mom likes to use ** to heat milk.	metal products
吸收很多热量会让**中心变成火红色。	He wrote an article introducing the uses of ** in the kitchen.	metal products
在古代，女子作为**需要负责管理日常生活。	In ancient times, women as ** needed to be responsible for managing daily life.	female occupations
一本历史小说中的人物曾经成为**处理日常安排。	A character in a historical novel once became ** handling daily arrangements.	female occupations
小说里描写一位**曾为国家带来许多和平。	The novel describes a ** who once brought much peace to the country.	female occupations
在戏剧中演员扮演**赢得了观众的喜爱。	In the drama, the actor playing ** won the audience's love.	female occupations
同学们通过学习知道**曾是古代不能缺少的官员。	Through studying, students learned that ** was once an indispensable official in ancient times.	female occupations
古代的书中多次提到**与日常生活管理制度密切相关。	Ancient books mentioned many times that ** was closely related to the daily life management syst	female occupations
观众很喜欢电视剧里**为百姓服务的样子。	The audience really likes the way ** serves the people in the TV series.	female occupations
历史课上同学们讨论**这一古代官位。	In history class, students discussed ** as an ancient official position.	female occupations
演讲比赛中有人讲过**如何收获百姓支持的故事。	In the speech competition, someone told a story about how ** gained the people's support.	female occupations
奶奶小时候听过**从天上飞下来的传说。	Grandma heard the legend of ** flying down from the sky when she was little.	female occupations
书里写到**拥有美丽和善良的心。	The book writes that ** possesses beauty and a kind heart.	female occupations
神话故事中说**居住在天上的云里。	Mythology says that ** resides in the clouds in the sky.	female occupations
小女孩把床单围在身上扮演**出现在朋友面前。	The little girl wrapped a bedsheet around herself to play ** and appeared in front of her friends.	female occupations
游客会去爬有**形象存在的山。	Tourists will go climb the mountain where the image of ** exists.	female occupations
有些人希望**能带来好运和幸福。	Some people hope that ** can bring good luck and happiness.	female occupations
古代传说中记载**能控制风和雨。	Ancient legends record that ** can control wind and rain.	female occupations
人们觉得小红像**一样美丽善良。	People think Xiao Hong is as beautiful and kind as **.	female occupations
姑娘们希望自己能像**一样优秀受人喜爱。	The girls hope they can be as excellent and beloved as **.	female occupations
草原上常常有一群**在奔跑。	On the grassland, there is often a group of ** running.	mammals
科学家发现**会亲自养大自己的宝宝。	Scientists have discovered that ** will personally raise their own babies.	mammals
刚出生的宝宝如果没有**保护会被其他动物吃掉。	Newborn babies will be eaten by other animals if they don't have ** protection.	mammals
无论来到哪里，每一只**都会在新地点留下自己的气味。	Wherever they go, each ** will leave its own scent at the new location.	mammals
农村附近常有**在寻找食物。	Near the countryside, there are often ** searching for food.	mammals
森林里有些**会吃掉植物的种子。	In the forest, some ** will eat plant seeds.	mammals
动物园里的大部分**都不怕人。	Most ** in the zoo are not afraid of people.	mammals
山顶上有一只**正在大叫向家人传递信息。	On the mountaintop, there is a ** calling loudly to transmit information to its family.	mammals
很多动物见到**都会选择远远地躲开。	Many animals will choose to stay far away when they see **.	mammals

科学家通过相机拍到**活动的珍贵画面。	Scientists captured precious footage of ** activities through cameras.	mammals
头上大大的角是**用来保护自己的武器。	The large horns on the head are weapons used by ** to protect themselves.	mammals
我们在森林里看到**吃树叶来获得营养。	We saw ** eating leaves to obtain nutrition in the forest.	mammals
教授说每年**都会长出新的角。	The professor said that every year ** will grow new horns.	mammals
有人在公路上碰见**带着自己的宝宝过马路。	Someone encountered ** taking its baby across the road on the highway.	mammals
论文里说有些**会长得比小汽车大很多。	The paper says some ** can grow much larger than a small car.	mammals
在冬天大部分**会不吃不喝睡三个月。	In winter, most ** will sleep for three months without eating or drinking.	mammals
在秋天的草原上很难找到**在哪里，因为它们跟土地颜色相似。	On the autumn grassland, it's hard to find where ** are, because they are similar in color to the lanc	mammals
古代人会把**皮制作成衣服。	Ancient people would make ** skin into clothing.	mammals
